# Optimal enumeration of state space of finitely buffered stochastic molecular networks and exact computation of steady state landscape probability

**DOI:** 10.1186/1752-0509-2-30

**Published:** 2008-03-29

**Authors:** Youfang Cao, Jie Liang

**Affiliations:** 1 Shanghai Center for Systems Biomedicine (SCSB), Shanghai Jiao Tong University, Shanghai, 200240, China; 2 Department of Computer Science and Engineering, Shanghai Jiao Tong University, Shanghai, 200240, China; 3 Department of Bioengineering, University of Illinois at Chicago, Chicago, IL 60612, USA

## Abstract

**Background:**

Stochasticity plays important roles in many molecular networks when molecular concentrations are in the range of 0.1 *μ*M to 10*n*M (about 100 to 10 copies in a cell). The chemical master equation provides a fundamental framework for studying these networks, and the time-varying landscape probability distribution over the full microstates, *i.e*., the combination of copy numbers of molecular species, provide a full characterization of the network dynamics. A complete characterization of the space of the microstates is a prerequisite for obtaining the full landscape probability distribution of a network. However, there are neither closed-form solutions nor algorithms fully describing all microstates for a given molecular network.

**Results:**

We have developed an algorithm that can exhaustively enumerate the microstates of a molecular network of small copy numbers under the condition that the net gain in newly synthesized molecules is smaller than a predefined limit. We also describe a simple method for computing the exact mean or steady state landscape probability distribution over microstates. We show how the full landscape probability for the gene networks of the self-regulating gene and the toggle-switch in the steady state can be fully characterized. We also give an example using the MAPK cascade network. Data and server will be available at URL: .

**Conclusion:**

Our algorithm works for networks of small copy numbers buffered with a finite copy number of net molecules that can be synthesized, regardless of the reaction stoichiometry, and is optimal in both storage and time complexity. The algorithm can also be used to calculate the rates of all transitions between microstates from given reactions and reaction rates. The buffer size is limited by the available memory or disk storage. Our algorithm is applicable to a class of biological networks when the copy numbers of molecules are small and the network is closed, or the network is open but the net gain in newly synthesized molecules does not exceed a predefined buffer capacity. For these networks, our method allows full stochastic characterization of the mean landscape probability distribution, and the steady state when it exists.

## Background

Networks of interacting biomolecules are at the heart of the regulation of cellular processes, and stochasticity plays important roles in many networks, including those responsible for gene regulation, protein synthesis, and signal transduction [1-5]. The stochasticity originates intrinsically from the small copy numbers of the molecular species in a cell, which frequently occur when molecular concentrations are in the range of 0.1 *μ*M to 1*n*M (typically from about 100 to 10 copies in a cell) [2,6]. For example, the regulation of transcriptions depends on the binding of often a few proteins to a promoter site; the synthesis of protein peptides on ribosome involves a small copy number of molecules; and patterns of cell differentiation depend on initial small copy number events. In these biological processes, fluctuations due to the stochastic behavior intrinsic in low copy number events play important roles.

The importance of stochasticity in cellular functions is well recognized. Studies of network models show that stochasticity is important for magnifying signal, sharpening discrimination, and inducing multistability [4,7-13]. Understanding the stochastic nature and its consequences for cellular processes involving molecular species of small copy numbers in a network is an important problem.

A fundamental framework for studying the full stochasticity is the chemical master equation [14,15]. Under this framework, the combination of copy numbers of molecular species defines the microscopic state of the molecular interactions in the network. By treating microscopic states of reactants explicitly, linear and nonlinear reactions (such as synthesis, degradation, dimeric binding, and multimerization) can all be effectively modeled as transitions between microstates, with transition rates determined by the physical properties of the molecules and the cell environment. The probability distribution or potential landscape [16-18] over these microstates and its time-evolving behavior provide a full description of the properties of a stochastic molecular network.

However, it is challenging to study a realistic system that involves a nontrivial number of species of small copy numbers. Analytical solutions of the chemical master equation exist only for very simple cases, such as self-regulating genes [19], and the toggle-switch network under certain restrictions [8,18]. Instead of solving the master equation, a widely used method is to carry out Monte Carlo simulations using the Gillespie algorithm [14]. This method generates samples from multiple runs of simulation, and statistics properties are calculated from the simulation trajectories, which provide characterizations of the network [13,14,20,21]. This approach has found wide applications, although it cannot guarantee a full account of stochasticity, as this method usually does not generate an exhaustive number of trajectories that cover all possible locations in the probability landscape. In addition, as Monte Carlo simulations follow high probability paths, it is especially challenging to sample adequately rare and critical events that may be important in determining cellular fate. It is also difficult to determine whether a simulation is extensive enough to obtain accurate statistics. The amount of computation necessary to obtain an accurate result may be too large to be completed in a reasonable amount of time, especially when the time scales of the various react ions involved are very different [8]. To address these issues, Gillespie, Petzold, and colleagues further developed numerical methods for speeding up the stochastic simulation [20,21]. Munsky and Khammash developed a method to approximate the solution of chemical master equation by projecting the whole state space of the system to a finite space [22]. Samant and Vlachos developed a multiscale Monte Carlo method for stiff systems where partial equilibrium occurs [23]. An alternative approach is to approximate the master equation using, for example, Fokker-Planck or Langevin equations [15]. These are obtained by adding stochastic terms (often Gaussian) to a deterministic equation [12,18,24]. Salis and Kaznessis improved the stochastic simulation method by partitioning the system into components with fast and slow reactions. The fast reactions are approximated by the Langevin equations, and the slow reactions are analyzed by stochastic Monte Carlo simulations [25].

A complete identification and characterization of the space of the microstates is a prerequisite for obtaining the full landscape probability distribution of a network. However, the state space of a network currently cannot be fully characterized in general. There is neither closed-form solution, nor computational algorithm describing the full state space. In this paper, we study the problem of enumerating the state space of a molecular network with small copy numbers of molecular species.

A naive method is to predefine the maximum copy number of the reactants, and bound the state space by the product of the maximum numbers. However, the size of state spaces estimated by this naive approach will be inflated to enormity. For example, if there are 16 species, and there is a total a maximum of 33 molecules in the whole system, this naive method does not take into consideration of the details of the network, and the state space will be estimated to be in the order of (33 + 1)^16 ^= 3.19 × 10^24 ^states. This naive method is intrinsically inefficient: There may be many states which may never occur. For some states, no reactions may occur and therefore are not needed. For others, no reactions can lead to them under the specified initial condition. An alternative approach is carrying out simulation. One can simply follow explicitly simulated reaction events to whatever microstates of copy numbers the system reaches. However, this approach cannot guarantee that all reachable states will be explored, therefore cannot guarantee full characterization of rare events.

In this study, we develop an optimal algorithm that gives full description of the state space and the set of transitions. Our method works for networks of small copy numbers under the condition that the net gain in newly synthesized molecules in the network does not exceed a predefined finite number. Our algorithm is optimal in both memory requirement and in time complexity. All states reachable from a given initial condition will be accounted for by our method, and no irrelevant states will be included. All possible transitions will be recorded, and no infeasible transitions will be ever attempted. As a result, our algorithm can generate the full state-transition matrix under the framework of the chemical master equation. This matrix is compact without any redundant information. It is also of the minimal size. In addition, the computational time is optimal, up to a constant. We also describe how to obtain the mean landscape probability distribution over the enumerated state space of a network, which is the same as the landscape distribution of the steady state when it exists.

This paper is organized as follows. We first describe how our method can be applied to the simple examples of a self-regulating gene, a toggle-switch network, and the more complex example of the MAPK network. This is followed by conclusion and discussion. We finally describe the technical details of the algorithm for enumerating the space of microstates, and introduce a simple method for computing the steady state landscape probability distribution.

## Results and Discussion

### Molecular network models

We apply our algorithm to three network models: the self-regulating gene, the small toggle-switch network, and the MAPK cascade network.

#### Self-regulating gene

**Figure 1:**
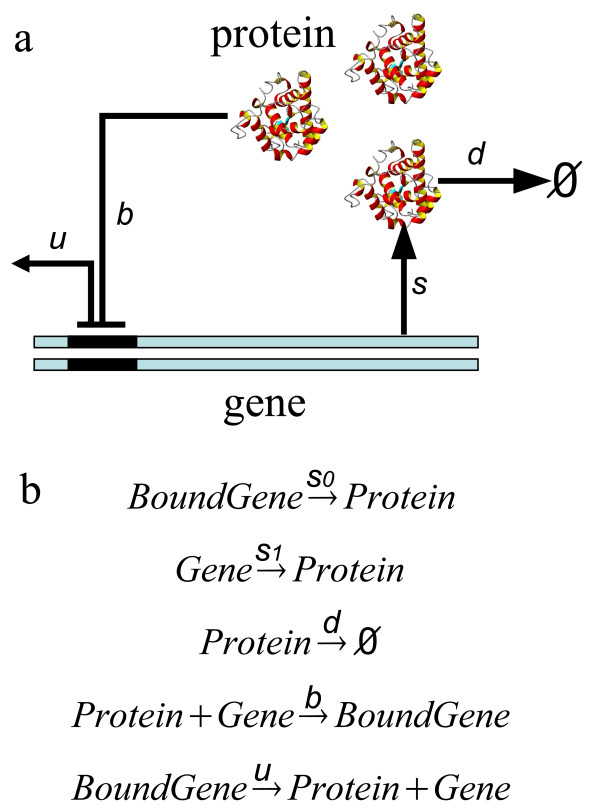
The network of a self-regulating gene. (a) The topology of the network. A single copy of the gene in the chromosome encodes a protein transcription factor (TF), which is synthesized at the rate of *s*_0 _or *s*_1_, depending on whether the operator site is bound (state 0) or unbound (state 1). The TF binds the operator site of the gene at a rate of *b*. It unbinds at a rate of *u*. The TF is also subject to degradation at a rate of *d *determined by the degradation machinery. Here the symbol ∅ represent the state of being degraded. (b) The chemical reactions of the five stochastic processes and the corresponding reaction rates.

Regulating the expression of even a single gene is a complex process. We study the network of an idealized self-regulating gene (Fig 1a and 1b). As a basic unit in biological genetic networks, it consists of only one gene, and is the simplest molecular network. We follow the study of Schultz *et al *and assume that the dominant form of regulation is the binding and unbinding of transcription factors to the operator site, which changes the rate of transcription initiation [18]. In this model, there are several stochastic processes: the synthesis and degradation of the protein transcription factor at the reaction rate constants of *s*_0 _(or *s*_1_) and *d*, respectively, and the binding and unbinding of the operator site of DNA by the transcription factor at the reaction rate constants of *b *and *u*, respectively. These processes are illustrated in Fig 1b. The binding state of the operator site is either "on/unbound" (state 1), or "off/bound" (state 0). The synthesis rate of transcription factor is either *s*_0 _or *s*_1_, depending on the binding state of the operator site.

We first calculate the state spaces. We use the same initial condition of 1 copy of unbound gene, 0 copies of transcription factor and bound gene, and set the buffer size to allow different copy numbers of protein transcription factor to be synthesized. As there is only one copy of the gene in this model [18], the size of the state space increases with the copy number of the protein transcription factor that can be synthesized. Our results show that when the buffer capacity takes the value of 100, 1,000, and 10,000, the size of the state space is 201, 2,001, and 20,001, respectively. In this model, the size of the state space scales linearly with the copy number of the protein synthesized. In biological condition, the copy number of a transcription factor rarely exceeds 100.

**Figure 2:**
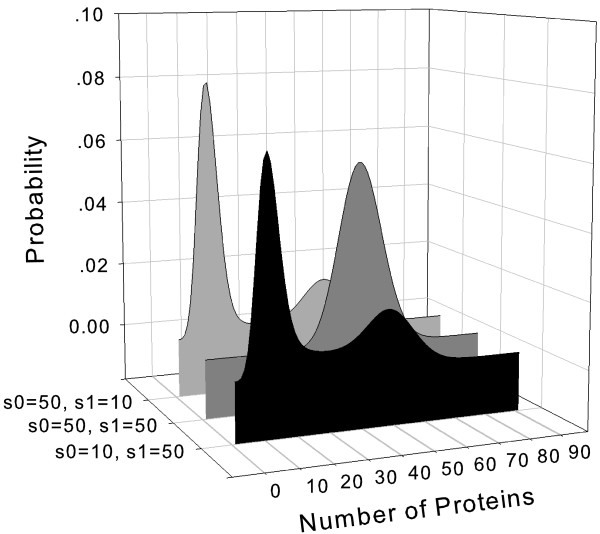
The steady state landscape probability distributions of a self-regulating gene network. The probability over the number of free protein is plotted. Here this probability is the sum of probabilities for two different gene binding states (bound and unbound) at the same number of free proteins. When the unbound/on state synthesis rate *s*_1 _is greater, the network is self-repressing. When the bound/off synthesis rate *s*_0 _is greater, the network is self-activating. Although the self-repressing (front profile) and the self-activating (back profile) genes have overall similar distributions, the former has a slightly higher probability in producing more free proteins than the latter. When both synthesis rates are equal (middle profile), the network follows a simple birth/death process, with a Gaussian probability distribution.

We then calculate the exact steady state probability distribution over the microstates of the self-regulating gene, namely, the exact steady state density function of different states of copy numbers of the transcription factor. In our calculation, the parameter values are chosen as *u *= *d*/10 and *b *= *d*/250, in units of degradation rate *d*, following reference [18]. The steady state distributions ***P ***at different values of synthesis rates in on/unbound and off/bound states *s*_1 _and *s*_0 _are computed exactly and are shown in Fig 2 for the case of buffer size of 1,010 for illustration. Here the marginal probability of having a specific number of free proteins in the system is plotted, regardless whether the gene is in off/bound or in on/unbound state. Following reference [18], we use three different network conditions: (*s*_0_, *s*_1_) = (50, 10), (50, 50), and (10, 50) in units of degradation rate *d*, respectively. When the on/unbound state synthesis rate *s*_1 _is greater, the network is self-repressing. When the off/bound synthesis rate *s*_0 _is greater, the network is self-activating.

Our results and the results of Schultz *et al *obtained from multiple runs of Gillespie simulations are identical [18]. As pointed out in [18], the self-repressing and the self-activating genes can have overall similar distributions. This can be explained by the fact that the combined synthesis rate of the protein *s*_0 _+ *s*_1 _= 60 is the same in both cases (front profile and back profile in Fig 2). Closer examination shows that in the case of the self-repressing gene network (*s*_0 _= 10 and *s*_1 _= 50, front profile), the first peak of probability at smaller copy number of the free protein is lower, and the second peak at higher copy number is larger when compared to the distribution of the self-activating gene (*s*_0 _= 50, and *s*_1 _= 10, Fig 2, back profile). That is, the self-repressing network has a higher probability in producing more free proteins than the self-activating network. This can be explained by the difference between the protein-DNA binding rate *b *and unbinding rate *u*. In this model network, unbinding rate *u *= *d*/10 is 25 times greater than the binding rate *b *= *d*/250. As a result, this gene is more likely to stay in the unbound state. Since the self-repressing network has a higher synthesis rate in unbound state (*s*_1 _= 50 > *s*_0 _= 10), it will produce more free proteins. This results in an overall slightly higher probability for larger number of free proteins for self-repressing network. This small difference in probability distribution is also observed in [18]. As pointed out previously in [18], when both synthesis rates are equal (*s*_0 _= *s*_1 _= 50), the binding state transition do not change the synthesis/degradation process, and the network is a simple birth/death process, with a Gaussian probability distribution for protein number centered at *s*_0 _= *s*_1 _(Fig 2, middle profile).

#### Toggle switch

**Figure 3:**
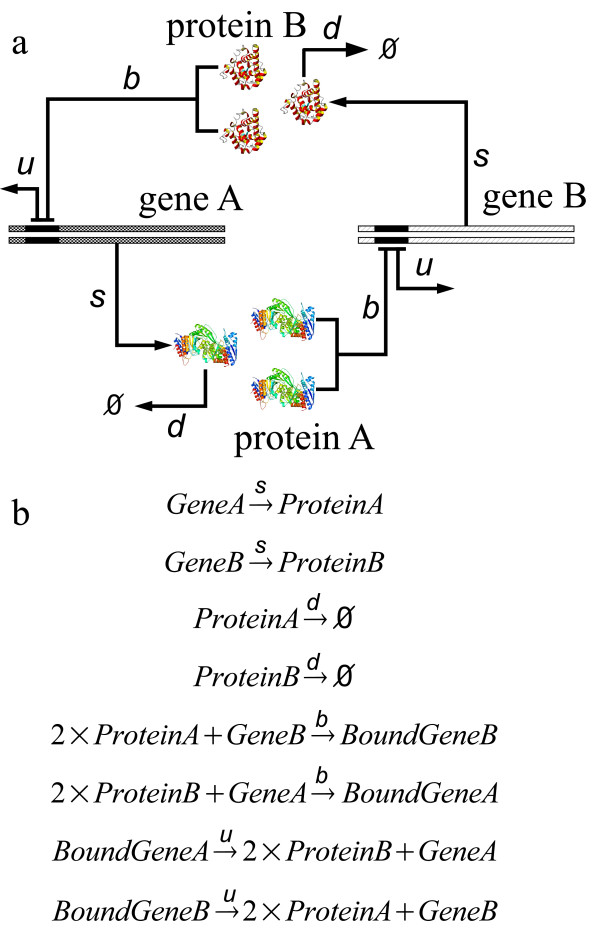
The network of a toggle switch. (a) The topology of the network and variables representing the reaction rates. Single copies of gene A and gene B in the chromosome each encode a protein product. Two protein monomers can repress the transcription of the other gene. The synthesis of protein product of gene A and B depends on the bound or unbound state of the gene. (b) The chemical reactions of the 8 stochastic processes involved in the toggle-switch network. The reaction rates include *s *for protein synthesis, *d *for protein degradation, *b *for protein-gene binding, and *u *for protein-gene unbinding.

A toggle switch is a small network consisting of two genes, A and B. The protein product of each represses the other gene. Toggle switch is the smallest genetic network that can present bistability. The insightful study of Schultz *et al *provided detailed analysis of the stochastic behavior of this model network [18]. To facilitate direct comparison, we adopt the same toggle-switch model developed by these authors (Eqns 5–8 in reference [18]). The molecular species and the network topology are shown in Fig 3a. There are a number of stochastic processes: the synthesis and degradation of proteins A and B, with reaction constants denoted as *s *and *d*, respectively; the binding and unbinding of the operator site of one gene by the protein products of the other gene at rate *b *and *u*, respectively (Fig 3b). The binding states of the two operator sites are "on-on/unbound-unbound" (state 11 for gene A and gene B), "on-off/unbound-bound" (state 10), "off-on/bound-unbound" (state 01), and "off-off/bound-bound" (state 00). The synthesis rates of both proteins A and B depend on the binding state of the operator sites. The toggle switch model used in this study and all possible chemical reactions in the model are extracted directly from the master equations in [18]. In this model, no dimerizations are explicitly modeled, and the model assumes that binding of two proteins to the operator site simultaneously. This is a valid approximation when the dimerization reaction is fast compared to all other reactions [8]. Even for this simple network, except for the special cases when "fast transition" between on- and off- operator states and "small noise" of high molecular concentration conditions are assumed, no exact solutions are known [8,18].

We first calculate the state spaces under the initial condition of 1 copy of unbound gene A, 1 copy of unbound gene B, 0 copies of bound gene A and bound gene B, and 0 copies of their protein products. We set the buffer size to different copies of total protein A and protein B combined that can be synthesized. When the buffer capacity is 20, the size of the state space is 764. At buffer capacity of 200, 400, and 800 copies of proteins, the size increases to 79,604, 319,204, and 1,278,404, respectively.

**Figure 4:**
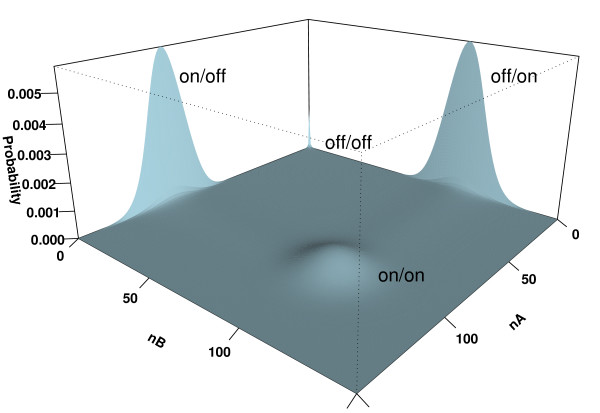
The steady state probability landscape of a toggle switch. A toggle switch has four different states, corresponding to different binding state of genes A and B. At the condition of small value of *u*/*b*, the off/off state is strongly suppressed, and the system exhibits bi-stability.

We then calculate the exact steady state landscape probability of the toggle-switch network, namely, the exact steady state density function of different microstates of copy numbers of products of gene A and gene B. The steady state distributions ***P ***are shown in Fig 4 for the case of buffer size of 300. In our calculation, the parameter values are chosen as *s *= 100*d*, *u *= *d*/10, and *b *= *d*/100, 000, in units of degradation rate *d*. These are the same as those used in reference [18].

It is clear that a toggle switch has four different states, corresponding to the "on/on", "on/off", "off/on" and "off/off" states. At the chosen parameter condition, the toggle/switch exhibits clear bi-stability, namely, it has high probabilities for the "on/off" and "off/on" states, but has a low probability for the "on/on" state. The "off/off" state is severely suppressed. Our results are identical with the results of Schultz *et al *obtained from multiple runs of Gillespie simulations [18].

#### MAPK network

MAPK cascade network plays important role in signal transduction. Here our purpose is to explore how to apply our algorithm to more realistic network model. Our goal in this paper is not to study the the stochastic nature and the dynamic behavior of MAPK network.

**Figure 5:**
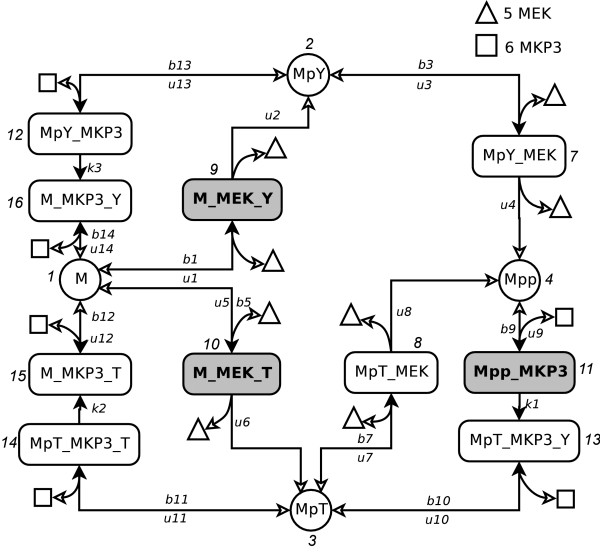
The MAPK network model according to BioModel (id BIOMD28). The molecular species are labeled with integer numbers. Reactions are labeled with variables representing the corresponding reaction rate, *b*_*i *_for binding rates, *u*_*i *_for unbinding rates, and *k*_*i *_for rates of first order reactions. Solid arrows in this figure represent binding reactions, and empty arrows for unbinding reactions. The parameter values of this model are taken as is from the SBML model. We have: *b*_1 _= 0.005, *b*_3 _= 0.025, *b*_5 _= 0.05, *b*_7 _= 0.005, *b*_9 _= 0.045, *b*_10 _= 0.01, *b*_11 _= 0.01, *b*_12 _= 0.0011, *b*_13 _= 0.01, *b*_14 _= 0.0018, *u*_1,3,5,7,9,10,11,13 _= 1, *u*_2 _= 1.08, *u*_4 _= 0.007, *u*_6 _= 0.008, *u*_8 _= 0.45, *u*_12 _= 0.086, *u*_14 _= 0.14, *k*_1 _= 0.092, *k*_2 _= 0.5, and *k*_3 _= 0.47.

The MAPK cascade network (BioModels ID: BIOMD0000000028) is taken from the BioModels database at EBI [26,27]. The molecular species and reactions are extracted from the SBML (Systems Biology Markup Language) model file. This network contains 16 molecular species with 17 reactions [26]. As there is no synthesis reaction, this particular network model is a closed system. Abbreviations used in this model are listed in Table 1. Fig 5 shows the topology of the model. All 16 molecular species are labeled with numbers from 1 to 16. Among them, MEK (triangles in Fig 5) and MKP3 (squares) are the key enzymes catalyzing all phosphorylation and dephosphorylation reactions in this network. The rest of the molecular species are substrates, intermediates, and products of MEK and MKP3 induced reactions. Most of the reactions in this model (14 of 17) are second-order.

**Table 1:** Abbreviations of the molecular species in the MAPK network.

Num.	Abbrev.	Description
1	M	ERK, extracellular signal- regulated kinase
2	MpY	ERK with Y phosphorylated
3	MpT	ERK with T phosphorylated
4	Mpp	ERK with dual phosphorylated
5	MEK	ERK kinase
6	MKP3	ERK phosphatase
7	MpY_MEK	Binding of MpY and MEK
8	MpT_MEK	Binding of MpT and MEK
9	**M_MEK _Y**	**Binding of M and MEK at Y site**
10	**M_MEK_T**	**Binding of M and MEK at T site**
11	**Mpp_MKP3**	**Binding of Mpp and MKP3**
12	MpY_MKP3	Binding of MpY and MKP3
13	MpT_MKP3_Y	Binding of MpT and MKP3 at Y
14	MpT_MKP3_T	Binding of MpT and MKP3 at T
15	M_MKP3_T	Binding of M and MKP3 at T site
16	M_MKP3_Y	Binding of M and MKP3 at Y site

#### Simple initial conditions

**Figure 6:**
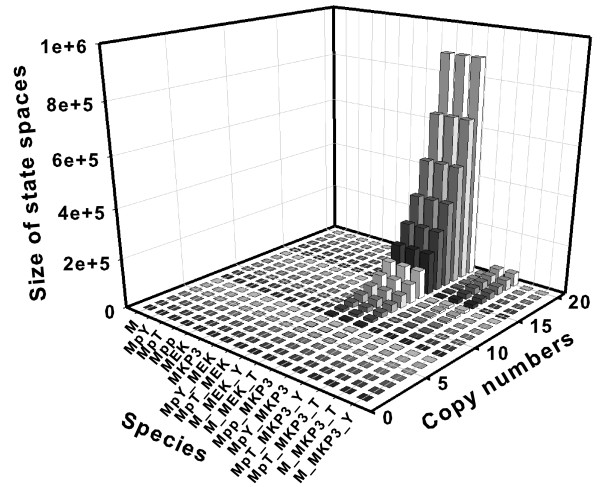
Sizes of state spaces for a model of the MAPK cascades under the initial condition of 1 to 20 copies of each of the 16 species in turn and 0 in all other species. Altogether the size of state space for 16 × 20 = 320 initial conditions are shown here.

We generate the state spaces of the MAPK network for different initial conditions and record their sizes. We first increase the copy number for one species from 1 to 20, and record the size of resulting state space, while keeping the copy numbers of all other species to 0. We repeat this process for each of the 16 molecular species in turn. Altogether, we have 16 × 20 = 320 data points of sizes of the state space (Fig 6).

It is clear that different molecular species in this model affect the size of the state space differently. Increasing the copy number of M-MEK-Y, M-MEK-T, and Mpp-MKP3 molecules (species 9, 10 and 11, in bold fonts in Table 1) lead to large state spaces (size 888, 030 at 20 copies, Fig 6), while the initial conditions of 20 copies of any other species result in modest state spaces. For example, species 7, 8, 15 and 16 when given 20 copies have a state-space size of 231. For species 1–6 (M, MpY, MpT, Mpp, MEK, MKP3), no reactions can occur at these initial conditions, and the state space contains only the the initial state.

The state space for each of the 320 initial conditions can be computed within one minute. We further found that when any of *S*_9_, *S*_10_, or *S*_11 _has an initial copy of 28 and all others 0 copies, the state spaces increases to 6,724,520, and the computing time also increase, although all can be computed within 10 minutes on a Linux workstation.

#### Biological initial conditions

We further calculate sizes of the state spaces with several biologically plausible initial conditions, in which species M, MEK and MKP3 are all given an equal number of *i *copies, while all the other species start with zero copies. We increase *i *from 1 to 11. These initial conditions correspond to a total number ranging from 3 × 1 = 3 copies to 3 × 11 = 33 copies of molecules of three species in the network. The size of the state space increases with the copy numbers. When there are 1 copy of M, MEK, and MKP3 each, the size of the state space is 14. For 5, 10, and 11 copies of M, MEK, and MKP3 each, the size increases to 8,568, 1,144,066, and 2,496,144, respectively. The computation of the state space at *i *= 10 and *i *= 11 requires 156 seconds and 589 seconds of CPU time on a Linux desktop machine, respectively.

#### Steady state distribution

We compute the steady state probability distributions of the microstates of the MAPK network at the initial condition of 10 copies each of M, MEK and MKP3. That is, we obtain the exact steady state density function of different microstates of all possible 1,144,066 combinations of different copy numbers of the 16 molecular species in the MAPK network. The computation is efficient. At this initial condition, the dimension of the Markovian transition matrix *M *is 1, 144, 066 × 1, 144, 066, with 14, 574, 406 number of non-zero elements. It takes 1,341 seconds (about 23 minutes) of CPU time to compute the steady state probability distribution on a Linux workstation.

**Figure 7:**
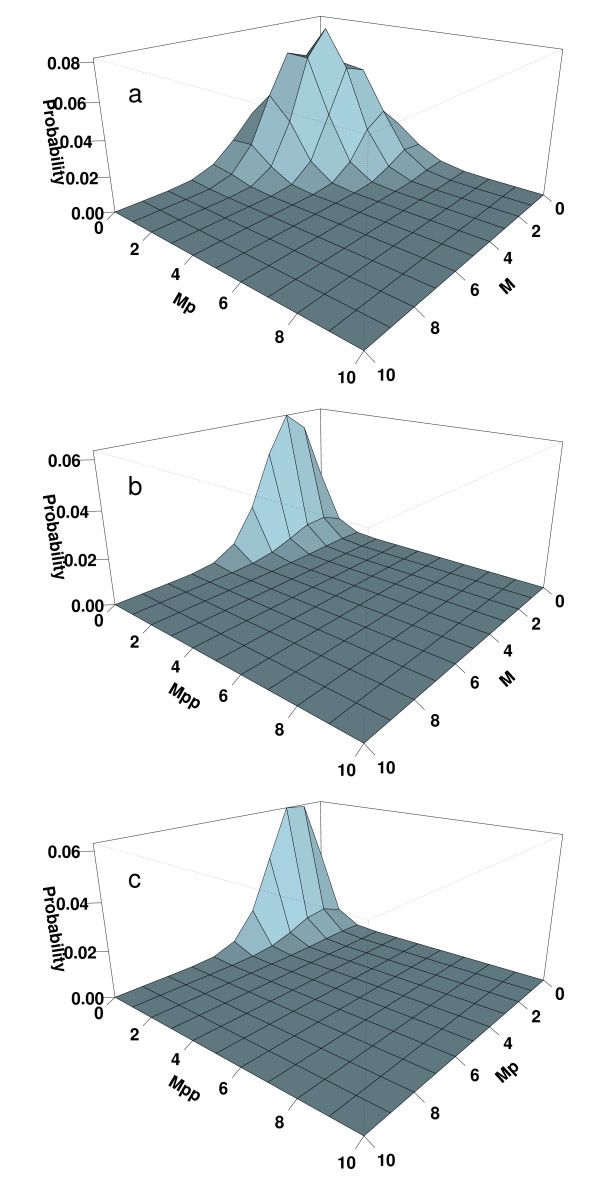
The marginal landscape probability distribution of different copy numbers of molecular species in the MAPK network in steady state. (a) Marginal probability distribution of the combination of the number of unphosphorylated ERK (M) and uniphosphorylated ERK (Mp, including both MpY and MpT), regardless of the copy numbers of all other molecular species; (b) Marginal probability distribution of the combination of the copy numbers of unphosphorylated ERK (M) and dual-phosphorylated ERK (Mpp); (c) Marginal probability distribution of the combination of the copy numbers of uniphosphorylated Mp and dual phosphorylated Mpp.

As it is impossible to directly visualize the landscape density distribution in a 16-dimensional space, for ease of visualization, we plot the marginal distribution of different combinations of copy numbers of extracellular signal-regulated kinase (ERK) in unphosphorylated state, in single phosphorylated state, and in dual phosphorylated state. Specifically, we plot the marginal probabilities of different copy numbers of unphosphorylated ERK (M), and ERK with either Y or T site phosphorylated (Mp, including both MpY and MpT), after integrating different copy numbers of all other 14 molecular species in Fig 7a. We plot the marginal distribution of different copy numbers of unphosphorylated ERK (M), and ERK with both Y or T site phosphorylated (Mpp) Fig 7b. We plot the marginal distribution of different copy numbers of uni-phosphorylated ERK with either Y or T site phosphorylated (Mp, including both MpY and MpT), and ERK with both Y or T site phosphorylated (Mpp) in Fig 7c. At this parameter condition, the steady state distribution has a single peak centered around two copies of unphosphorylated ERK (M), two copies of uni-phosphorylated ERK (Mp), and zero copy of dual phosphorylated ERK (Mpp).

## Conclusion

Stochasticity plays important roles in molecular networks for processes involving small copy numbers of molecules. Models of molecular networks based on macroscopic reaction rates and coupled ordinary differential equations are not applicable in these cases, as they can only model high concentrations of interacting molecules with negligible fluctuations.

The stochastic nature of molecular interactions at low copy numbers can be fully characterized if the time-varying landscape probability distribution on all of the microstates of a molecular network can be computed. This is a difficult task, as the state space of the combination of the copy numbers of molecular species needs to be explicitly enumerated, the probability distribution over these microstates and changes of this distribution across many decades of time scale need to be fully computed.

In this study, we have developed an algorithm to enumerate the state space of a molecular network of small copy numbers with a buffer containing a finite number of molecules that can be synthesized. It can also be used to find all possible transitions between states, and to compute the transition rates between these states. We also demonstrate how to obtain the steady state probability distribution based on the enumerated states when it exists.

Our example of the toggle-switch network shows that this method can be used to study the rise of important network properties such as bistability. The enumeration of the full state space of the MAPK cascade network at various initial conditions demonstrate that our method can be used to study a realistic network of nontrivial size, which is more complicated than the simple networks that are often studied for full stochasticity. Although naively the state space at the initial condition of each of 11 copies of unphosphorylated, uniphosphorylated, and biphosphorylated ERK kinase might be as high as (33 + 1)^16 ^= 3.19 × 10^24^, a truly astronomical size, our method showed that the relevant space is only about 2.50 × 10^6^, which is amenable for computation using a desktop computer.

Our method is applicable to study various carefully constructed model network systems. It complements the Monte Carlo simulation method, as it can be used to characterize the full probability landscape of networks with enumerable state space. For example, it will allow the calculation of the probabilities of the occurrence of rare and critical events. For theoretical studies, one can predefine a fixed number of net molecules that can be synthesized, and investigate the nature of the landscape probability distribution. This is similar to the studies of semi-grand canonical ensemble in statistical physics [28]. Exact characterization of probability landscape is useful, as most network studies are based on stochastic simulation, and relative little is known at the level of the full stochastic landscape probability distribution, even for simple toy systems. For example, analytical solutions to the simple toggle switch model is known only when the model parameters follows the restrictions of small noise and fast transition [8,18]. We believe our method can be used to study well designed model systems beyond self-regulating genes and simple toggle switches, and the exact results obtained will be helpful for understanding the basic properties and design principles governing stochastic networks. A useful analogy to illustrate the utility of such model studies can be found in the field of protein folding, where a large number of studies using simple short chain HP lattice models revealed remarkable insights about how complex proteins fold [29-36].

Our method can also be applied to more realistic biological networks, such as the MAPK network model, which is a closed system according to the annotated BioModels database [27]. Such closed systems could arise when one focus on a submodule of a larger network. For the majority of realistic networks which are open systems, an important determining factor of the applicability of our method is the limit of the capacity of a buffer, which has to be greater than the maximum copy number of the net gain in protein molecules that can be synthesized. In a cell, this maximum number is determined by the life time of the cell, and the net synthesis rate of protein molecules. The latter depends on both protein synthesis and degradation rates. A simple approach is to estimate the net number of protein molecules that can be synthesized during the life time of a cell. For example, the lifespan of an *E. coli *cell is about 30 minutes [37]. Estimation based on the rate limiting processes of transcription initialization and elongation indicate that the protein synthesis rate ranges from 0.0077/*s *(for the C1 protein) [2,38] to 0.0534/*s *for the Cro protein [2,39,40] in the lambda phage system. Their degradation rates are about 0.0007/*s *and 0.0025/*s*, respectively [2]. This suggests that a useful bound of the copy number of newly synthesized molecules for studying the lambda switch network system could be in the order of 150–200 copies under reasonable initial conditions. Naturally, the exact number will depend on the details of the chosen network model and the parameter values. For example, models of cells under stress with retarded synthetic rates may require a relatively small buffer capacity.

In this study, we have described a method to compute the steady state landscape probability distribution. Steady state distribution is of general interests when it exists, as has been shown in previous studies [17,18]. For realistic network, another approach is to compute the time-dependent dynamic change of landscape probability distribution, using techniques such as those used in [36]. We will describe this approach in more details in future studies.

As the number of molecular species and their copy numbers increase, the state space will eventually become prohibitively large for explicit computation even with an optimal algorithm. In these cases, our method can be used to select important states and to control error bounds at a specific tolerance for developing approximation methods, an approach well demonstrated in [22].

## Methods

### The Algorithm

Suppose we have a model of a biological network, which contains *m *molecular species and can have *n *reactions. Given an initial condition, namely, the copy numbers of each of the *m *molecular species, we aim to calculate all states that the biological system can reach starting from this initial condition, under the condition that the net number of molecules that can be synthesized does not exceed a predefined limit. These states collectively constitute the state space of the network under this initial condition.

Formally, we have a model of a biological network ***N ***= **(*M***, ***R***), with *m *+ 1 number of molecular species: ***M ***= (*M*_1_,...*M*_*m*+1_), and *n *reactions: ***R ***= {*R*_1_,..., *R*_*n*_}. Here *m *of the species are from the network. A buffer of predefined capacity is used to represent a pool of virtual molecules for open systems, from which synthesis reactions can generate new molecules, and to which degradation reactions can deposit molecules removed from the network. We use the *m *+ 1-th species to represent this buffer pool. The combinations of copy numbers of all molecular species ***S ***= (*c*_1_,...,*c*_*m*_, *c*_*m*+1_) form the microstate of the system, where *c*_*m*+1 _denotes the number of net new molecules that can still be synthesized at this state. A reaction can involve an arbitrary number (≥ 1 and ≤ *m*) of molecular species as reactants and/or products, with any arbitrary positive integer coefficient (*i.e*., arbitrary stoichiometry). Synthesis reaction is allowed to occur only if the buffer pool is not exhausted, namely, only if *c*_*m*+1 _> 0. The set of all possible states ***S ***that can be reached from an initial condition following these rules constitute the state space of the system: X = {**S**}. The set of allowed transitions is ***T ***= {*t*_*ij*_}. We are given with an initial condition: $S^{t=0}=(c_{1}^{0},c_{2}^{0},...,c_{m}^{0},c_{m+1}^{0})$, where $c_{i}^{0}$ is the initial copy number of the *i*-th molecular species at time *t *= 0, and $c_{m+1}^{0}=B$ is the predefined buffer size. The maximum copy number of net gain in newly synthesized molecules of the system is restricted by this constant *B*. Our aim is to enumerate the state space X under this given initial condition.

The algorithm is written as Algorithm 1 (see Appendix). It performs the following computation: After initialization, we start with the initial state *S*^*t *= 0^. We examine each reaction in turn to determine if this reaction can occur for the current state. If so, and if the buffer is not exhausted, we generate the state that this reaction leads to. If the newly generated state was not encountered before, we add it to our collection of states for the state space, and declare it as a new state. We repeat this for all new states, which is maintained by a stack data structure. This terminates when all new states are exhausted.

In this algorithm, a stack data structure is used. Description of the stack data structure can be found in computer science textbooks such as [41]. A stack is used here to store individual states. These states are "Push"ed onto the stack: If we encounter a previously unseen state, we create it and push it onto the stack so further calculations on this state can be carried out at a later stage. We use the "Pop" operation to obtain a state previously stored on the stack to carry out these calculations. In this case, we pop a state to examine what reactions can occur and what other states these reactions can lead to.

We can compute the transition coefficient {*a*_*i*, *j*_} between two microstates ***S***_*i *_and ***S***_*j *_using Algorithm 2 (see Appendix) following the approach outlined in references [14,18,22]. We give further details in later sections on how this is done for the three networks studied here.

#### Correctness and optimality

The state space and the transitions under a given initial condition can be considered as a directed graph *G*= (X, ***T***), in which vertice are the state vectors, *i.e*., the set of reachable states X, or the *m *+ 1-tuples of copy numbers of the *m *+ 1 molecular species, including the buffer. Edges are the set of allowed transitions ***T ***between the states, *i.e*., reactions connecting two state vertice. Two vertice ****S***_*i *_*∈ X and ***S***_*j *_∈ X are connected by a directed edge *t*_*i*, *j *_∈ ***T ***if and only if ***S***_*i *_can be transformed to ***S***_*j *_through a reaction. Any reachable state can be transformed from the initial state by one or more steps of reactions, and the directed graph *G *is a connected graph.

Our algorithm implicitly generates this graph *G*. Because the set of reactions ***R ***is finite, *G *has a finite tree-width at any finite steps away from the initial condition. Assume the algorithm will not terminate in finite steps. Since in this algorithm each state is only visited no more than twice, *G *must have an unlimited depth. That is, there must exist a path *p *in the graph *G *that starts from the initial state and extends to infinite. Therefore *p *must contain an infinite number of different states. This is impossible for any given initial condition, as each molecular species has a limited initial copy number, and the size of the buffer limits the number of new molecules that can be synthesized in open systems. The algorithm therefore must terminate.

This algorithm gives correct answers, assuming that the newly synthesized molecules does not exceed the predefined buffer capacity. This is because all states visited in the algorithm can be reached from the initial condition, and all visited states is actually reached as each is brought to by a chemical reaction. In addition, all reachable states will be visited, as the algorithm test at each state all possible reactions, and will only terminates when all new states are exhausted. It is easy to see all possible transitions between states will be recorded.

The time complexity of our algorithm is optimal. Since only unseen state will be pushed onto the stack, every state is pushed and popped at most once, and each state will be generated/visited at most twice before it is popped from the stack. As access to each state and to push/pop operations take *O*(1) time, the total time required for the stack operations is *O*(|X|). As the algorithm examines each of the *n *reactions for each reached state, the complexity of total time required is *O*(*n*|X|), where *n *is usually a modest constant (e.g. < 50). Based on the same argument, it is also easy to see that the algorithm is optimal in storage, as only valid states and valid transitions are recorded.

#### Computing mean and steady state probability distribution

We can calculate the expected landscape probability distribution over the microstates, namely, the exact mean density function of different microstates of copy numbers in the network. It is the same as the steady state probability distribution function if the steady state exists. Instead of calculating the time trajectories of changes in the probability distribution and wait until it reaches equilibrium, we use a simpler approach applicable to networks in which a steady state exists. Following Kachalo *et al *[36], we obtain the Markovian state transition matrix ***M ***from the reaction rate matrix ***A***: ***M ***= ***I ***+ ***A***·Δt, where ***I ***is the identity matrix, and Δ*t *is the discrete time unit and is chosen to be 1. The probability distribution function ***P ***of the microstates can be obtained by solving the equation ***P ***= ***MP***. The calculation of the steady state distribution ***P ***is not sensitive to the precise choice of the discrete time unit Δ*t*. The steady state distribution corresponds to the eigenvector of ***M ***with eigenvalue of 1. We use the Arnoldi method implemented in the software ARPACK to compute the steady state distribution ***P ***[42].

### Computing transition coefficients

The transition coefficient between different states connected by a reaction is calculated by multiplying the intrinsic rate of this reaction with the reaction order dependent combination number of copies of reactants in the "before" state [14]. We provide more details using examples from the three networks.

#### Self-regulating gene

Suppose the first order reaction

$$Protein\overset{d}{\to }\emptyset$$

enables the transition of the system from the microstate *i *to *j*. This reaction denotes the degradation of the protein molecule at an intrinsic rate of *d*. The stoichiometry of this reaction dictates that the copy number of protein *n*_*p*, *j *_in the "after" state *j *is one less than the copy number *n*_*p*, *i *_in the "before" state *i*. From the reaction formula, the transition coefficient *a*_*i*, *j *_for the matrix **A **is calculated as:

*a*_*i*, *j *_= *d·n*_*p*, *i*_.

Recall that since a microstate is uniquely determined by the combination of copy numbers of all molecular species, *n*_*p*, *i *_therefore is known as a state attribute.

For the second order reaction

$$Protein+Gene\overset{b}{\to }BoundGene,$$

the transition coefficient connecting the "before" state *i *to the "after" state *j *can be computed as:

*a*_*i*, *j *_= *b*·*n*_*p*, *i*_·*n*_*g*, *i*_,

where *b *is the intrinsic reaction rate, *n*_*p*, *i *_is the protein copy number at state *i*, and *n*_*g*, *i *_is the copy number of gene in state *i*, which is 1, as we assume there is only one copy of the gene in this network model.

We can similarly compute the transition coefficient *a*_*i*, *j *_for the reaction

$$BoundGene\overset{u}{\to }Protein+Gene$$

as *a*_*i*, *j *_= *u*·*n*_*bg*, *i*_, where *n*_*bg*, *i *_is the number of bound gene in the "before" state, which takes the value of 0 or 1 in this model, depending on whether the gene is in protein-free or in protein-bound state. For the simpler reaction:

$$BoundGene\overset{s_{0}}{\to }Protein,$$

we have *a*_*i*, *j *_= *s*_0_·*n*_*bg*, *i*_, where *n*_*bg*, *i *_is the number of bound gene in state *i*, which takes the value of 0 or 1. For the synthetic reaction of

$$Gene\overset{s_{1}}{\to }Protein,$$

we have *a*_*ij *_= *s*_1_·*n*_*g*, *i*_.

We have described how to compute the transition coefficient for all reactions in the represser gene network. In Algorithm 2, we can compute the transition coefficient *a*_*i*, *j *_based on the formula of the reaction leading from state *i *to state *j*.

#### Toggle switch

For the third order reaction

$$2\times ProteinA+GeneB\overset{b}{\to }BoundGeneB,$$

the transition coefficient *a*_*i*, *j *_can be computed as

*a*_*i*, *j *_= *b*·*n*_*gB*, *i*_·*n*_*pA*, *i*_·(*n*_*pA*, *i *_- 1)/2,

where *b *is the intrinsic reaction rate, *n*_*pA*, *i *_is the copy number of protein A in state *i*, and *n*_*gB*, *i *_is the copy number of unbound gene *B*, which takes the value of 0 or 1. For this third order reaction, the number of possible ways of choosing two protein molecules from *n*_*pA*, *i *_copies is $\left(\begin{array}{c} n_{pA,i}\\ 2 \end{array}\right)=n_{pA,i}\cdot (n_{pA,i}-1)/2$. Transition coefficients for the other reactions in this network can be computed similarly following this reaction and the reactions described earlier for the represser gene network.

#### MAPK network

We consider the second order binding reaction

$$M+MKP3\overset{b_{14}}{\to }M\_MKP3\_Y.$$

If the "before" state *i *is transformed to the "after" state *j *by one step of this reaction, the corresponding transition coefficient *a*_*i*, *j *_can be computed as

*a*_*i*, *j *_= *b*_14_·*n*_*M*, *i*_·*n*_*MKP*3, *i*_

where *b*_14 _is the intrinsic reaction rate, *n*_*M*, *i *_and *n*_*MKP*3, *i *_are the copy numbers of M and MKP3 molecules in state *i*, respectively. The other transition coefficients in this network can be computed similarly using the intrinsic reaction rates given in Fig 5 and the copy numbers of reactants determined by the "before" state *i*.

## Authors' contributions

JL designed the algorithm for state space enumeration and steady state computation. YC and JL designed the method for transition matrix generation, YC implemented these algorithms, and developed the molecular models. YC carried out computation. YC and JL analyzed the data and wrote the paper together.

## Appendix

**Algorithm 1 **State Enumerator(***M***, ***R***, *B*)

Network model: ***N ***← {***M***, ***R***};

Initial condition: $S^{t=0}\leftarrow \{c_{1}^{0},c_{2}^{0},...,c_{m}^{0}\}$; Set the value of buffer capacity: $c_{m+1}^{0}\leftarrow B$;

Initialize the state space and the set of transitions: X ← ∅; ***T ***← ∅;

Stack *ST *← ∅; Push(*ST*, ***S***^*t *= 0^); *StateGenerated *← FALSE

**while ***ST *≠ ∅ **do**

   ***S***_*i *_← Pop (*ST*);

   **for ***k *= 1 to *n ***do**

      **if **reaction *R*_*k *_occurs under condition ***S***_*i *_then

         if reaction *R*_*k *_is a synthetic reaction and generates *u*_*k *_new molecules **then**

            *c*_*m*+1 _← *c*_*m+1 *_- *u*_*k*_

            **if ***c*_*m*+1 _≥ 0 **then**

               Generate state ***S***(*i*, *R*_*k*_) that is reached by following reaction *R*_*k *_from ***S***_*i*_;

               *StateGenerated *← TRUE

            **end if**

         **else**

            **if **reaction *R*_*k *_is a degradation reaction and breaks down *u*_*k *_molecules **then**

               *c*_*m*+1 _← *c*_*m*+1 _+ *u*_*k*_

            **end if**

            Generate state ***S***(*i*, *R*_*k*_) that is reached by following reaction *R*_*k *_from ***S***_*i*_;

            *StateGenerated *← TRUE

         **end if**

         **if **(*StateGenerated *= TRUE) and (***S***(*i*, *R*_*k*_) ∉ X) **then**

            X ← X ∪ ***S***(*i*, *R*_*k*_);

            Push(*ST*, ***S***(*i*, *R*_*k*_));

            ***T ***← ***T ***∪ $t_{S_{i},S(i,R_{k})}$;

            *a*_*i*, *j *_← Transition Coefficient(***S***_*i*_, ***S***(*i*, *R*_*k*_), *R*_*k*_)

         **end if**

      **end if**

      **end for**

end while

Output X, ***T ***and ***A ***= {*a*_*i*, *j*_}.

**Algorithm 2 **Transition Coefficient(***S***_*i*_, ***S***_*j*_, *R*_*k*_)

Read in reaction rate parameters for *R*_*k*_

Retrieve the copy numbers of molecular species occurring in the reaction formula of *R*_*k *_from the state vector ***S***_*i*_

Compute the combination copy numbers of each reactant molecular species

Compute transition coefficient *a*_*i*, *j *_based on the reaction rate parameters for *R*_*k*_, and the combination copy numbers.

Output *a*_*i*, *j*_.
